# Quantitative microbial risk assessment of antibiotic resistance genes and mobile genetic elements in orchard soils across South Korea

**DOI:** 10.1128/aem.02260-25

**Published:** 2025-12-18

**Authors:** Raan Shin, Seunggyun Han, Jaeyoung Ro, Sujin Lee, Song-Hee Ryu, Hor-Gil Hur, Hanseob Shin

**Affiliations:** 1 School of Environment and Energy Engineering, Gwangju Institute of Science and Technology (GIST) 65419, Gwangju, South Korea; 2 Residual Agrochemical Assessment Division, National Institute of Agricultural Sciences 230986, Wanju-gun, South Korea; 3 Center for Health Effects of Environmental Contamination, University of Iowa 4083, Iowa City, Iowa, USA; 4 State Hygienic Laboratory, University of Iowa 160410, Coralville, Iowa, USA; Michigan State University, East Lansing, Michigan, USA

**Keywords:** antibiotic resistance genes, orchard soils, agricultural antibiotics, quantitative microbial risk assessment, occupational exposure

## Abstract

Antibiotic resistance is a global health crisis, but environmental pathways of resistance dissemination to farm workers remain poorly understood. Agricultural soils represent critical but underexplored reservoirs of antibiotic resistance genes (ARGs) and mobile genetic elements (MGEs), particularly in orchards where antibiotics such as streptomycin and oxytetracycline are widely used for fire blight control. Here, we conducted a nationwide investigation of orchard soils in South Korea, integrating high-throughput qPCR, 16S rRNA gene sequencing, and quantitative microbial risk assessment (QMRA). We detected 297 ARGs and 52 MGEs, with eight core genes [*aac(3)-VIa*, *tetL*, *aadE*, *sul1*, *qacH_351*, *tnpA-1*, *IS*6100, and *intI1*] significantly enriched in orchard soils but absent in non-orchard soils, such as national parks or mountain soils. Aminoglycoside- and tetracycline-resistance genes were dominant, directly reflecting the application of streptomycin and oxytetracycline. QMRA estimated that orchard farmers ingest resistance genes through soil contact, with *aac(3)-VIa* posing the highest risk (~29 ingestion events per farmer annually), followed by *qacH_351*, *tetL*, and *tnpA-1*. These results demonstrate the quantifiable occupational risks of ARG exposure in orchard environments. By combining resistome profiling, microbial networks, and QMRA, this study establishes a framework for assessing the public health implications. Although the ingestion of ARGs may not immediately cause impacts on human health, such exposure has the potential to enrich antibiotic resistance within the gut microbiome of farm workers, thereby increasing the probability of treatment complications if infections occur.

**IMPORTANCE:**

Antibiotic resistance is widely recognized as one of the most concerning threats to public health, yet the pathways through which resistance emerges and spreads remain underexplored. Orchard soils, where antibiotics are sprayed to control plant diseases, represent an overlooked environment where resistance may develop and circulate to people who work the land. By examining soils from orchards at a nationwide scale, we found resistance genes that mirror the antibiotics used in these settings and showed that farm workers are regularly exposed to them through routine contact with soil. This study provides the direct evidence that orchard farming can contribute to human exposure to resistance, heralding the need to include agricultural environments in efforts to prevent the spread of resistance. Our work offers a way to measure these risks and can guide protective strategies for workers and communities.

## INTRODUCTION

The rising use of antibiotics significantly contributes to the emergence and spread of antibiotic resistance (AR), posing a growing threat to both human and animal health (1). Despite these concerns, the development and continued use of antibiotics remain indispensable for modern medicine. In addition to their clinical applications, antibiotics are widely consumed in agriculture for crop protection, animal husbandry, and livestock production (2). It has been well documented that agricultural antibiotic usage has substantially contributed to the proliferation of antibiotic resistance genes (ARGs) (3). Moreover, beyond the direct use of antibiotics, agricultural practices such as manure and biosolid application have markedly increased the prevalence of ARGs in soils and water, thereby facilitating the persistence and dissemination of antibiotic-resistant bacteria (ARB) (2, 4). For instance, elevated ARG levels have also been observed in greenhouse soils following the manure application (5).

In orchard systems, antibiotics have become essential for disease control, particularly against fire blight, a bacterial disease severely affecting apple and pear trees. In South Korea, streptomycin, oxolinic acid, and oxytetracycline are routinely applied during the blooming season to suppress bacterial infections (6), which occur more frequently in orchards compared to other agricultural crop systems (7). Such prolonged and repeated applications may accelerate the emergence of ARB (8). Furthermore, continuous agricultural inputs could promote the enrichment and mobilization of ARGs through mobile genetic elements (MGEs), increasing the potential for transmission via pathogens and subsequent exposure to humans, particularly orchard workers (9).

Quantitative microbial risk assessment (QMRA) is needed to protect the health of workers in agricultural environments. Since risk assessment estimates the likelihood of disease caused by infections, the widespread use of antibiotics calls attention to the urgent need to evaluate human exposure to antibiotic resistance in the environment (10). Effective risk assessment requires the identification and continuous surveillance of ARGs (11, 12). Then, QMRA provides a structured framework to quantify potential exposure to antibiotic resistance in specific environments and to evaluate the associated health risks (13). A study in the United States assessed the risk of ARG and MGE exposure through contaminated wells (13), while another study in India used QMRA to evaluate the risk of exposure to β-lactam resistance genes in water and street food (14). Despite increasing concerns, systematic risk assessment has not been conducted in agricultural environments in South Korea.

Thus, in South Korea, where antibiotics are widely applied in orchards, understanding the dynamics of ARGs is essential for QMRA to protect agricultural workers. We conducted a nationwide assessment of ARGs and MGEs in orchard soils and further applied QMRA to evaluate potential exposure risks to workers. This study could provide critical risk assessment information on the potential human health impacts associated with ARG exposure in agricultural environments.

## RESULTS AND DISCUSSION

### Resistome and mobilome analysis in orchard samples

**Fig 1:**
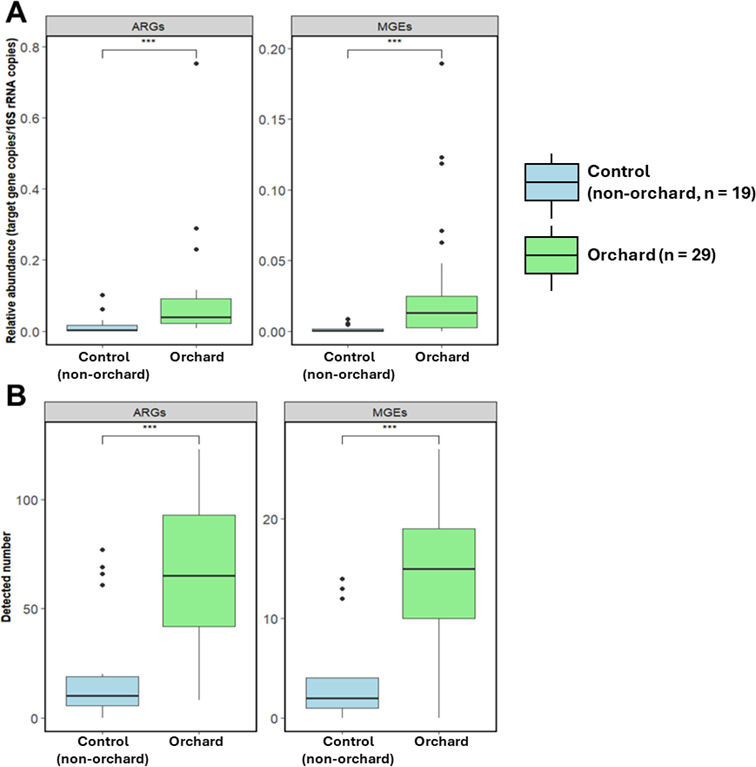
Comparison of the abundance and diversity of antibiotic resistance genes (ARGs) and mobile genetic elements (MGEs) between orchard (*n* = 29) and control (non-orchard, *n* = 19) samples. (**A**) Relative abundance of ARGs and MGEs in orchard and control samples. (**B**) Detected numbers of ARGs and MGEs in orchard and control samples. Statistical significance was determined using the Wilcoxon test. ***P < 0.001.

**Fig 2:**
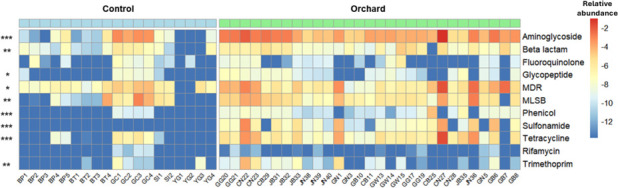
A heatmap for the distribution of ARG types in orchard and control samples. The legend values represent log_10_ (relative abundance, target gene copies/16S rRNA gene copies). In the legend, red and blue indicate higher and lower relative abundance, respectively. Green and blue bars above the heatmap indicate orchard and control samples, respectively. Statistical significance was determined using the Wilcoxon test. *P < 0.05, **P < 0.01, ***P < 0.001. MDR, multidrug resistance.

A total of 297 ARGs and 52 MGEs were identified in orchard soil samples. The relative abundances of ARGs and MGEs were significantly higher in orchard soils compared to controls (*P* < 0.001, Fig. 1A). Similarly, the number of detected ARGs and MGEs was also significantly greater in orchard samples than in controls (*P* < 0.001, Fig. 1B). In particular, with the exception of fluoroquinolone- and rifamycin-resistance genes, all other ARG classes showed significantly higher relative abundances in orchard samples compared to controls (*P* < 0.05, Fig. 2). Notably, aminoglycoside-resistance genes exhibited the highest enrichment across orchard soils, showing their dominant role in these environments (*P* < 0.001). Other ARG classes, including phenicol, sulfonamide, and tetracycline, were also significantly more abundant in orchard samples (*P* < 0.001). β-Lactam, trimethoprim, and macrolide-lincosamide-streptogramin (MLSB) resistance genes were moderately enriched (*P* < 0.01), while multidrug resistance and glycopeptide resistance genes were detected at significantly higher levels in orchard soils than in controls (*P* < 0.05).

These observations align with previous studies in agricultural environments, such as greenhouse soils, which also exhibited higher ARG diversity and abundance compared to non-agricultural soils (15). Furthermore, long-term agricultural activities, including manure application and antibiotic treatments, contributed to the enrichment and dissemination of ARGs by exerting selective pressure on soil microbiomes (5). In orchard systems, streptomycin (an aminoglycoside) and oxytetracycline (a tetracycline) are among the most frequently used antibiotics for the management of fire blight (9). The dominant enrichment of aminoglycoside-resistance genes and the significantly elevated levels of tetracycline-resistance genes observed in our orchard soils are therefore consistent with the types of antibiotics commonly applied in these environments. This suggests that the continued use of streptomycin and oxytetracycline in orchards may be a major driver of ARG persistence and proliferation, reflecting direct selective pressure on the soil resistome.

### Identification of core ARGs and MGEs in orchard soils

Linear discriminant analysis effect size (LEfSe) analysis identified eight core genes [five ARGs: *aac(3)-VIa*, *tetL*, *aadE*, *sul1*, and *qacH_351*, and three MGEs: *tnpA-1*, *IS*6100, and *intI1*] (Fig. S1A). Notably, a transposase gene, *tnpA-1*, was detected in 26 orchard samples, representing the most frequently identified core MGE, while *aac(3)-VIa* was detected in 28 orchard samples. Except for these two genes, the remaining six core genes (*tetL*, *aadE*, *sul1*, *qacH_351*, *IS*6100, and *intI1*) were absent in all control (non-orchard) soil samples. The concentration of *tnpA-1* 1 (0–0.099 copies per 16S rRNA gene copies) was the highest among MGEs and was significantly enriched in manure-amended soils compared to control soils (Fig. S1B).

These findings are consistent with previous evidence that *tnpA* proliferates in manure-treated agricultural environments (16). Likewise, the relative abundances of *tnpA* and *IS*6100 increased under high antibiotic concentrations, further supporting their responsiveness to anthropogenic selective pressure (17). Another MGE, *intI1*, also serves as a well-established indicator of ARG dissemination due to its key role in driving the horizontal transfer of multiple resistance determinants (11). Among ARGs, *aac(3)-VIa* exhibited the highest relative abundance (0–0.058 copies per 16S rRNA gene copy) across 28 orchard soil samples (Fig. S1B), suggesting its value as an indicator gene for monitoring ARG contamination in agricultural soils. Importantly, *aac(3)-VIa* is commonly carried on IncA/C plasmids and frequently associated with *intI1*, indicating a strong potential for horizontal gene transfer (HGT) (18). It has been reported in *Escherichia coli* from cattle feces and poultry, suggesting its mobility and dissemination across agricultural and environmental settings (18).

The aminoglycoside-resistance gene, *aadE*, has been detected in agricultural soils (19). Its presence in orchard soils is consistent with streptomycin use for fire blight management, indicating the selective pressure that drives the proliferation of aminoglycoside resistance in these environments. Similarly, the abundance of the tetracycline-resistance gene, *tetL*, has been shown to increase under oxytetracycline exposure and is frequently detected in *Enterococcus faecalis* from tetracycline-contaminated pig manure (20). The enrichment of *tetL* in orchard soils thus reflects the intensive use of oxytetracycline in fruit production. Together, the detection of *aadE* and *tetL* establishes a direct link between agricultural antibiotic usage (streptomycin and oxytetracycline) and the dominance of aminoglycoside- and tetracycline-resistance genes in orchard soils.

The sulfonamide resistance gene, *sul1*, has been frequently reported in agricultural environments (21), while *qacH_351*, a quaternary ammonium compound resistance gene, has been enriched in hospital wastewater and urban rivers (22). Moreover, *qacH_351* abundance increased under polymyxin B treatment, suggesting that antibiotic pressure may further contribute to its environmental proliferation.

These findings demonstrate that the identified core genes in orchard soils serve as potential indicator genes for monitoring AR contamination. The enriched aminoglycoside- and tetracycline-resistance genes, in particular, herald the direct impact of streptomycin and oxytetracycline application in orchards, emphasizing the role of antibiotic pressure in shaping the soil resistome.

### Bacterial community structure in orchard soils

**Fig 3:**
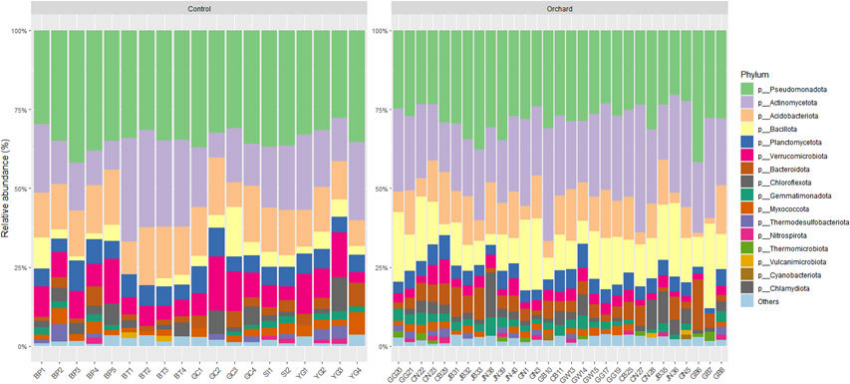
Stacked bar plots for the relative abundance of bacterial phyla (>1% of total community) across orchard and control soil samples. The left and right panels represent control and orchard soils, respectively. Phyla with <1% relative abundance are grouped as others.

Bacterial taxa in orchard and control soils were identified at the phylum level. The composition of major phyla (relative abundance >1%) is compared in Fig. 3. The distribution of the five dominant phyla differed markedly between orchard and control samples. In orchard soils, Pseudomonadota was the most abundant phylum, accounting for 19.9%–40.9% of the community, followed by Actinomycetota (13.4–38.2%), Bacillota (2.9%–25.3%), Acidobacteriota (1.3%–14.6%), and Bacteroidota (1.1%–15.7%) (Table S1). In contrast, the control soils were dominated by Pseudomonadota (23.3%–37.3%), Actinomycetota (6.54%–30.1%), Acidobacteriota (7.26%–17.6%), Verrucomicrobiota (3.1%–14.3%), and Planctomycetota (3.8%–8.5%) (Table S1). Statistical comparison revealed that Bacillota, Bacteroidota, and Actinomycetota were significantly more abundant in orchard soils than in controls (Wilcoxon matched-pair test, *P* < 0.05), whereas Pseudomonadota, Acidobacteriota, Verrucomicrobiota, and Planctomycetota were significantly enriched in control soils (*P* < 0.05) (Fig. S2).

Among the dominant phyla, Actinomycetota and Bacillota are strongly associated with the presence and mobility of ARGs (23). Previous studies have identified Bacillota as a dominant phylum under long-term antibiotic selection pressure (24). Members of Bacillota, particularly the genera *Streptococcus*, *Enterococcus*, and *Staphylococcus*, are known to be major contributors to the horizontal gene transfer of ARGs through plasmids and MGEs under antibiotic pressure (25). Similarly, Actinomycetota, characterized by their high G + C content (26), are well recognized for producing diverse bioactive metabolites, including antibiotics, anticancer compounds, and enzymes (27). Actinomycetes isolated from various soil environments often exhibit resistance to an average of seven to eight antibiotics, reflecting their intrinsic resistance mechanisms and adaptive strategies in competitive soil ecosystems (28).

**Fig 4:**
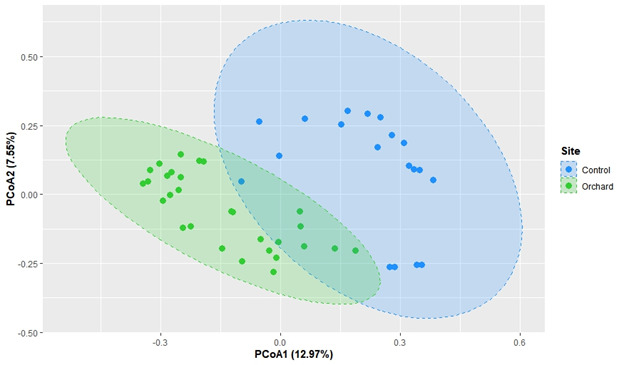
Principal coordinate analysis (PCoA) of bacterial community composition in orchard and control soils. PCoA based on Bray–Curtis distances was performed using permutational multivariate analysis of variance with adonis function in RStudio. Each point represents the bacterial community of an individual soil sample, with green and blue indicating orchard and control soil samples, respectively. Ellipses represent the 95% confidence intervals for each group. Orchard and control soils formed distinct clusters (*R*² = 0.13985, *P* < 0.001), indicating significant differences in community composition.

β-Diversity analysis further revealed that microbial communities from orchard soils clustered closely together while being clearly separated from control soils (*R*^2^ = 0.14, *P* < 0.001) (Fig. 4). LEfSe analysis identified 34 genera with LDA scores greater than 3 that were significantly enriched in orchard samples compared to controls (Fig. S3), indicating distinct microbial assemblages associated with agricultural environments. A detailed discussion of the bacterial community analysis is provided in the supplemental material.

### Relationships among bacterial community, ARGs, and MGEs

**Fig 5:**
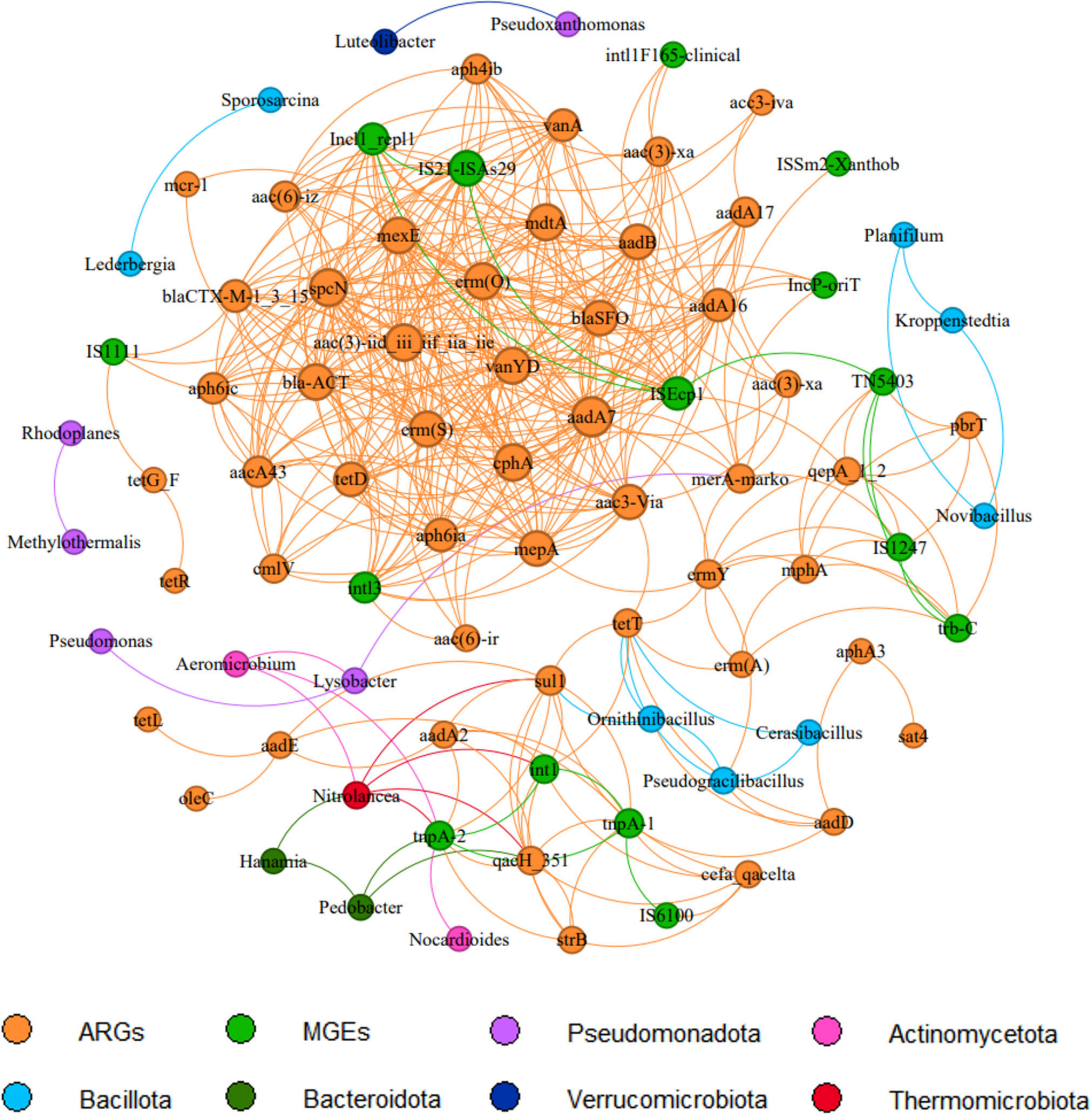
Network analysis linking antibiotic resistance genes (ARGs), mobile genetic elements (MGEs), and bacterial genus. Co-occurrence networks were constructed using Spearman’s correlation (*R* > 0.6, *P* < 0.05). Nodes represent ARGs (orange), MGEs (green), or bacterial genera grouped by phylum (each color is indicated in the legend). Edges denote significant positive correlations between nodes.

Eight bacterial hosts, potentially carrying ARGs and MGEs, were identified through network analysis based on Spearman correlations (Fig. 5). The network analysis generated 84 nodes and 430 edges, representing significant associations between bacterial genera, MGEs, and ARGs. The eight bacterial genera showed strong correlations with multiple ARGs (*ermY*, *tetT*, *aadD*, *sul1*, *merA*, and c*qacH_351*) and MGEs (*int1* and *tnpA-2*). Among these, *Nitrolancea* sp. exhibited the highest number of associations, being significantly correlated with four resistance-associated elements (*qacH_351*, *sul1*, *int1*, and *tnpA-2*), suggesting its potential importance as a key mediator in ARG dissemination. *Pseudogracilibacillus* sp. showed positive correlations with *ermY*, *tetT*, and *aadD*, while *Ornithinibacillus* sp. was associated with *tetT*, *aadD*, and a core gene, *sul1. Pedobacter* sp. was significantly correlated with *aadD* and another core gene, *qacH_351*. In addition, three genera, *Aeromicrobium* sp., *Cerasibacillus* sp., and *Nocardioides* sp., were significantly correlated with the mobile element *tnpA-2*, suggesting their potential involvement in transposon-mediated ARG transfer. Notably, *Pseudogracilibacillus* sp., which was not detected in the control samples, showed correlations with multiple ARGs.

A previous study reported that *Pseudogracilibacillus* sp. is capable of surviving under antibiotic-treated conditions and was able to persist even after the thermophilic composting phase (29). Furthermore, this genus has been identified as a dominant taxon in chicken manure compost (30), suggesting its strong ecological resilience in agricultural environments. Similarly, *Pedobacter* spp. are resistant to multiple antibiotics and have been predominantly found in an aminoglycoside-resistant bacterial isolate collection (31).

### Influence of soil physicochemical characteristics on bacterial communities and ARGs

**Fig 6:**
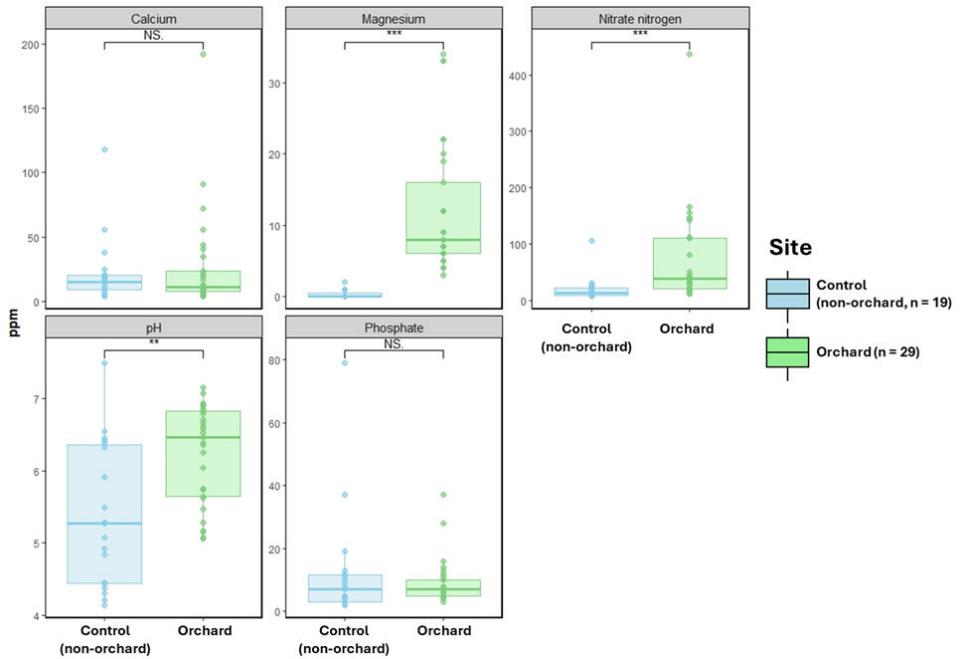
Comparison of the concentrations of four soil ions (nitrate, magnesium, calcium, and phosphate) and pH between orchard and control samples. Statistical significance was determined using the Wilcoxon test. **P < 0.01, ***P < 0.001.

Orchard soils exhibited distinct physicochemical characteristics compared to control soils, with significantly higher concentrations of nitrate (NO^3−^) and magnesium (Mg^2+^) (*P* < 0.001) and elevated pH (*P* < 0.01), while calcium (Ca^2+^) and phosphate (PO_4_^3−^) showed no significant differences (Fig. 6). Spearman correlation analysis further revealed that several enriched bacterial genera were significantly associated with soil parameters, with pH showing the strongest negative correlations. Notably, genera such as *Nitrolancea*, *Nocardioides*, and *Aeromicrobium* were positively correlated with NO_3_^−^ and Mg^2+^ and also linked to ARGs and MGEs (Fig. S4). Detailed analyses and discussion of these correlations are provided in the supplemental material.

### Risk assessment of core genes in orchard through QMRA

QMRA was conducted to assess the potential risks associated with the ingestion of ARGs and MGEs by orchard farmers. The analysis focused on unintentional ingestion of soil particles carrying resistance genes, with the objective of quantifying long-term exposure risks linked to core ARGs and MGEs. Considering gene ingestion as a direct exposure pathway, this study points out the importance of soil contact as a critical route for ARG transmission in agricultural environments (orchard in this study).

Among ARGs, *aac(3)-VIa* showed the highest daily ingested gene dose (1.99 copy numbers/day, 95% confidence interval [CI]: 1.3–3.06) and the highest daily probability of gene ingestion (0.52, 95% CI: 0.43–0.60) (Table 1). In terms of annual exposure, *aac(3)-VIa* also presented the highest risk, with an estimated 29.38 ingestion events per farmer per year (95% CI: 24.51–34.20) (Table 1). This was followed by *qacH_351* (10.20 cases/year), *IS*6100 (9.50 cases/year), *tetL* (8.78 cases/year), *intI1* (7.62 cases/year), *sul1* (6.57 cases/year), and *aadE* (5.38 cases/year). For MGEs, *tnpA-1* exhibited the highest estimated risk at 10.41 cases/year (95% CI: 5.70–16.53), followed by *IS*6100 and *intI1*. Such MGE-mediated genes represent significant pathways of potential exposure in orchard soils.

**TABLE 1:** Daily and annual estimated exposure of target genes among orchard farmers^*a*^

Target gene	Daily ingested gene dose, *D* (copy numbers/day, median [95% CI])	*P* (unitless, median [95% CI])	*E* (cases/year, median [95% CI])
*aac(3)-VIa*	1.99 (1.3–3.06)	0.52 (0.43–0.60)	29.38 (24.51–34.2)
*tnpA*	0.77 (0.26–3.26)	0.18 (0.10–0.29)	10.41 (5.7–16.53)
*tetL*	0.49 (0.17–1.73)	0.15 (0.08–0.25)	8.78 (4.56–14.25)
*IS*6100	0.43 (0.18–1.26)	0.17 (0.09–0.27)	9.5 (5.13–15.39)
*aadE*	0.17 (0.10–0.25)	0.09 (0.06–0.13)	5.38 (3.42–7.41)
*int1*	0.43 (0.13–1.63)	0.13 (0.07–0.22)	7.62 (4.0–12.54)
*sul1*	0.30 (0.11–1.00)	0.12 (0.06–0.20)	6.57 (3.42–11.4)
*qacH_351*	0.60 (0.18–1.81)	0.18 (0.08–0.29)	10.2 (4.56–16.53)

^*a*^ Median values with 95% confidence intervals (CI) were obtained from Monte Carlo simulations, assuming daily soil ingestion rates of 10–100 mg/day and average annual working days in orchards. Daily exposure doses (*D*, copy numbers/day) were calculated based on relative abundance data (target gene copies/16S rRNA gene copies) in soil. The daily probability of gene ingestion (*P*, unitless) was estimated using an exponential dose–response model. The annual exposure cases (*E*, cases/year) were derived by multiplying *P* by the annual number of working days. Median values with 95% CIs were obtained from Monte Carlo simulations, assuming daily soil ingestion rates of 10–100 mg/day and average annual working days in orchards. *D *values (copy numbers/day) were calculated based on relative abundance data (target gene copies/16S rRNA gene copies) and absolute 16S rRNA gene concentrations in soil.

Network-based correlation analysis (see Fig. 5) revealed strong associations between ARGs, MGEs, and specific bacterial genera, indicating that these taxa may act as carriers and facilitators of resistance dissemination. Given the frequent and unintentional soil contact by orchard farmers, identifying bacterial genera that harbor core ARGs is crucial for understanding potential transmission pathways of resistance. Although ingestion of ARGs and MGEs may not directly result in immediate illness, the possibility of HGT within the human gut microbiome poses an indirect significant public health concern (13). This study introduces a framework for evaluating ARG-related risks in orchard environments by integrating quantitative estimates of gene ingestion with microbial interaction networks, thereby improving understanding of how resistance may persist and spread in agricultural ecosystems.

### Conclusions

In this study, we applied QMRA to agricultural environments as a means to evaluate human health risks from ARGs and MGEs. While the clinical aspects of antibiotic resistance are well recognized, environmental exposures, particularly through direct soil contact in orchards, remain unexplored. Indeed, gene ingestion via soil represents an exposure pathway for orchard farmers, driving the need to integrate QMRA into environmental monitoring and management frameworks.

To fill this gap, we conducted a nationwide investigation of orchard soils in South Korea, identifying 297 ARGs and 52 MGEs, including eight core genes strongly linked to streptomycin and oxytetracycline use. Distinct microbial communities enriched in orchards were correlated with ARGs, MGEs, and soil physicochemical factors. QMRA revealed that aminoglycoside-resistance gene, *aac(3)-VIa*, posed the highest annual exposure risk (~29 ingestion events per farmer), followed by *qacH_351*, *tetL*, and *tnpA-1*. By integrating comprehensive approaches (resistome profiling, microbial network analysis, and QMRA), this study could establish a framework for quantifying ARG-related risks in agroecosystems and contribute to developing strategies to mitigate resistance dissemination and protect farmer health.

## MATERIALS AND METHODS

### Description of sampling sites and sample collection

A total of 29 orchard soil samples were collected in 2023 from eight provinces across South Korea. To provide a representative nationwide assessment, orchard sites were selected across South Korea, covering a broad range of regions. Orchard sites were selected based on the orchard database provided by the Rural Development Administration. Sampling was conducted in March, prior to the blooming season. During site visits, some orchards had already received manure or antibiotic applications, while others were in the process of applying them. For orchard sites that were no longer accessible at the time of sampling, nearby orchards were sampled after confirming with farmers that agricultural activities were being conducted. However, detailed information regarding the quantity and duration of antibiotic or fertilizer application was not available.

In addition, 19 non-orchard soil samples were collected as controls from mountainous areas and provincial parks that were not influenced by agricultural activities. Detailed information on all sampling sites is provided in Table S2. Soil samples from each site were collected as composite samples from a depth of 5–15 cm. All samples were transported to the laboratory under cold conditions and stored at −20°C until further analysis.

### Analysis of soil physicochemical parameters

The dried soils were sieved through a 2 mm mesh prior to downstream analyses. The concentrations of Mg^2+^, Ca^2+^, NO^3−^, PO_4_^3−^, and pH were measured using a Rapid-d PIA-001 ion analyzer (Technell, South Korea) following the manufacturer’s instructions. For pH determination, 5 g of soil was suspended in 25 mL of sterile distilled water, shaken for 1 h, and measured using a Star A211 Benchtop pH Meter (Thermo Fisher).

### DNA extraction

DNA was extracted from 0.5 g of soil using the DNeasy PowerSoil kit (Qiagen, Hilde, Germany) according to the manufacturer’s instructions. The concentration of the extracted DNA was measured using a Nanodrop spectrophotometer (MicroDigital Co., Korea). Extracted DNA samples were stored at −20°C for downstream analysis.

### High-throughput quantitative PCR of antibiotic resistance gene and mobile genetic elements

High-throughput quantitative PCR (HT-qPCR) was conducted using the SmartChip qPCR program (v.2.7.0.1; Wafergen Biosystem, USA) to quantify target genes in the samples. A total of 382 primers were designed and validated to amplify 319 ARGs, 57 MGEs, and 16S rRNA genes (32). The performance of the HT-qPCR array has been experimentally validated, showing amplification efficiency between 90% and 110%, detection limits as low as 1–10 gene copies/reaction, a linear dynamic range spanning approximately six to seven orders of magnitude, and standard curves with *R*^2^ > 0.99 (32). The 319 ARG primer sets include major antibiotic classes such as aminoglycosides, β-lactams, fluoroquinolones, glycopeptides, MLSB, phenicols, rifampicins, sulfonamides, multidrug ARGs, tetracyclines, and trimethoprim. For the 57 MGEs, insertional, integrase, plasmid, and transposase were included.

Each PCR mixture contains 1× LightCycler 480 SYBR Green I Master Mix (Roche Inc., Basel, Switzerland), 5 ng/µL of DNA template, 500 nM of reverse and forward primers (32), and nuclease-free PCR-grade water. Amplification was performed using the following protocol: initial denaturation at 95°C for 10 minutes, followed by 40 cycles of denaturation at 95°C for 30 seconds, and annealing at 60°C for 30 seconds. Melting curve analysis was provided using the SmartChip qPCR program (v.2.7.0.1, Wafergen Biosystem). All qPCR reactions were performed in triplicate, and only the positive runs in triplicates were used for analysis. Reactions with multiple melting peaks or amplification efficiencies outside the range of 1.8–2.2 were discarded. A threshold cycle of 31 was considered the detection limit. Gene copy numbers were calculated using the following equation (33): gene copy number = $10^{\left(31-CT\right)/\left(10/3\right)}$. The copy numbers of ARGs and MGEs were normalized by those of 16S rRNA to determine the relative abundance.

### Risk assessment

The QMRA approach was used to assess the risk associated with the potential ingestion of ARGs and MGEs by orchard farm workers. QMRA was conducted in the following sequence (34): (i) hazard identification, (ii) exposure assessment, (iii) dose–response assessment, and (iv) risk characterization.

#### Hazard identification

LEfSe algorithm was used to identify core genes (35) and to compare orchard soils with control (non-orchard) soils in South Korea. In this study, core genes were defined as ARGs and MGEs that were consistently and significantly enriched across multiple orchard soil samples relative to control soils. Core genes significantly enriched in orchard soils were considered indicators of resistance contamination in agricultural environments. Since ARGs and MGEs have the potential to pose health risks (13, 36, 37), they were defined as potential hazards in orchard soils in this study.

#### Exposure assessment

Exposure assessment was conducted to estimate the potential ingestion of ARGs and MGEs by farmers through soil contact. The model focused on unintentional ingestion of soil particles during agricultural activities. Based on similar agricultural environments (38), our study assumed that farmers ingest between 10 and 200 mg of soil per day in orchards. This range was determined from WHO and EPA guidelines, particularly EPA’s high-contact scenarios and soil-plus-dust ingestion rates for adults. The selected range (10–200 mg/day) reflects higher levels of soil exposure in labor-intensive agricultural settings, where farmers frequently come into contact with soil. The daily exposure dose (*D*, gene copy numbers/day) was calculated based on the gene copy numbers estimated from the relative abundance of target genes. It was obtained by multiplying the gene concentration in the soil (*C*, gene copy numbers/mg of soil) by the daily soil ingestion rate (*R*, mg/day), as shown in equation (1):


(1) D=gene concentrations in the soil (C)×daily ingestion rates of soil (R).

#### Dose–response assessment

The daily probability of gene ingestion (*P*, unitless) was estimated using an exponential dose–response model with a conservative coefficient *r* = 1^13^. Because specific dose–response parameters for ARGs and MGEs have not been experimentally validated, the assumption of *r* = 1 follows standard QMRA practice for highly uncertain agents. *P* was calculated following equation (2):


(2) P=1−exp(−D).

In this equation, *D* represents the daily dose of the gene (gene copy numbers/day) estimated in the exposure assessment, and *P* is the probability of the daily dose of gene ingested by farmers.

#### Risk characterization

Risk was characterized as the annual number of gene ingestion events for individual orchard farmers, as shown in equation (3). The parameters used in risk assessment were the previously calculated probability of the daily dose (*P*) and the estimated annual working days of orchard farmers (d/y) averaging about 50 days per year (range: 20–65 days) based on reported working times for major fruit crops (39). The estimated annual exposure cases (*E*, gene ingestion events/year) were calculated based on equation (3):


(3) Annual exposure cases=probability of the daily dose (P)×annual working days (d/y),

where *P* is the daily probability of gene ingestion, and the annual working days (d/y) represent the number of working days per year. Based on reported working times for major fruit crops (apple, pear, and grape) (39), orchard farmers worked on average 50 days per year (range: 20–65 days), consistent with typical orchard maintenance and harvest periods (40).

### Analysis of bacterial community using 16S rRNA gene sequencing

For bacterial community analysis, the hypervariable region (V3–V4) of the bacterial 16S rRNA gene was amplified with primers (341F: CCTACGGGNGGCWGCAG and 805R: GACTACHVGGGTATCTAATCC). Amplicon sequencing was performed using the Illumina Miseq platform. Amplicon sequence variants (ASVs) were generated following the DADA2 (v.1.18.0) pipeline (41), which included steps for error correction, merging, and denoising. Taxonomy classification was performed by classifying ASVs using the NCBI 16S reference database with a Bayesian classifier implemented in DADA2 (v.1.18.0) (42), and downstream analyses were conducted using QIIME (v.1.9.0) (43).

### Statistical analysis

Data analysis and calculations were performed by using R Studio (v.4.3.3) and Microsoft Excel 2020. The Wilcoxon test was applied to compare the relative abundances of ARGs, MGEs, ARG type, soil physicochemical parameters, and bacterial phyla between orchard and control samples, using the “ggsignif” package in R studio. Principal coordinate analysis based on Bray–Curtis distances was performed using the “vegan” package to determine the distribution of bacterial communities. Statistical significance of compositional differences was further evaluated with permutational multivariate analysis of variance using the adonis function in the vegan package. To identify core genes and bacterial genera enriched in orchard samples, the LEfSe algorithm was performed (44). The core bacterial genera identified through LEfSe were further used for network analysis and correlation with soil physicochemical parameters. Relationships between ARGs, MGEs, and the bacterial genera in orchard samples were analyzed through Spearman’s correlation. Based on Spearman coefficients (*R*^2^ > 0.63, *P* < 0.05), a co-occurrence network was constructed using Gephi.

QMRA was performed using two-dimensional Monte Carlo (2DMC) simulations, utilizing the “mc2d” package and the “fitdistrplus” package (45). The 2DMC approach separates uncertainty and variability. This study conducted 1,000 iterations in each dimension (total of 4,000,000 [2,000 uncertainty dimension × 2,000 variability dimension] Monte Carlo simulations). The gene concentration data (*C*) was modeled by fitting parametric distributions. *C* was assumed to be based on distributions defined by bootstrap procedure, reflecting uncertainty and variability. Daily ingestion rates of soil (*R*) were assumed to follow a uniform distribution (10–200 mg/day) to represent variability. The daily exposure dose (*D*) was simulated using a Poisson distribution based on the estimated exposure dose. Monte Carlo simulation outputs were summarized by calculating the arithmetic mean in the variability dimension, and then calculating the median and 95% confidence intervals across the uncertainty dimension.

## Data Availability

Raw sequencing data for each sample analyzed in this study have been deposited and are available under BioProject accession number PRJNA1320974.

## SUPPLEMENTAL MATERIAL

Supplemental figures and tables aem.02260-25-s0001.docx Figures S1 to S4; Tables S1 and S2.

Supplemental text aem.02260-25-s0002.docx Supplemental results.

ASM does not own the copyrights to Supplemental Material that may be linked to, or accessed through, an article. The authors have granted ASM a non-exclusive, world-wide license to publish the Supplemental Material files. Please contact the corresponding author directly for reuse.

## References

[1] Tiseo K, Huber L, Gilbert M, Robinson TP, Van Boeckel TP. 2020. Global trends in antimicrobial use in food animals from 2017 to 2030. Antibiotics (Basel) 9:1–14. doi:10.3390/antibiotics9120918 PMC7766021 33348801

[2] Mann A, Nehra K, Rana JS, Dahiya T. 2021. Antibiotic resistance in agriculture: perspectives on upcoming strategies to overcome upsurge in resistance. Curr Res Microb Sci 2:100030. doi:10.1016/j.crmicr.2021.100030 34841321 PMC8610298

[3] Wang F, Fu YH, Sheng HJ, Topp E, Jiang X, Zhu YG, Tiedje JM. 2021. Antibiotic resistance in the soil ecosystem: a One Health perspective. Curr Opin Environ Sci Health 20:100230. doi:10.1016/j.coesh.2021.100230

[4] Li Y, Kong F, Li S, Wang J, Hu J, Chen S, Chen Q, Li Y, Ha X, Sun W. 2023. Insights into the driving factors of vertical distribution of antibiotic resistance genes in long-term fertilized soils. J Hazard Mater 456:131706. doi:10.1016/j.jhazmat.2023.131706 37247491

[5] Fang H, Wang H, Cai L, Yu Y. 2015. Prevalence of antibiotic resistance genes and bacterial pathogens in long-term manured greenhouse soils as revealed by metagenomic survey. Environ Sci Technol 49:1095–1104. doi:10.1021/es504157v 25514174

[6] Ham H, Oh GR, Park DS, Lee YH. 2022. Survey of oxolinic acid-resistant Erwinia amylovora in Korean apple and pear orchards, and the fitness impact of constructed mutants. Plant Pathol J 38:482–489. doi:10.5423/PPJ.OA.04.2022.0059 36221920 PMC9561153

[7] Ha H-Y, Park S-E, You A-S, Gil G-H, Park J-E, Lee I-Y, Park K-W, Ihm Y-B. 2016. Survey of pesticide use in leaf and fruit vegetables, fruits, and rice cultivation areas in Korea. Weed Turfgrass Sci 5:203–212. doi:10.5660/WTS.2016.5.4.203

[8] McGhee GC, Sundin GW. 2011. Evaluation of kasugamycin for fire blight management, effect on nontarget bacteria, and assessment of kasugamycin resistance potential in Erwinia amylovora. Phytopathology 101:192–204. doi:10.1094/PHYTO-04-10-0128 20923369

[9] Martínez JL. 2008. Antibiotics and antibiotic resistance genes in natural environments. Science 321:365–367. doi:10.1126/science.1159483 18635792

[10] Ashbolt NJ, Amézquita A, Backhaus T, Borriello P, Brandt KK, Collignon P, Coors A, Finley R, Gaze WH, Heberer T, Lawrence JR, Larsson DGJ, McEwen SA, Ryan JJ, Schönfeld J, Silley P, Snape JR, Van den Eede C, Topp E. 2013. Human health risk assessment (HHRA) for environmental development and transfer of antibiotic resistance. Environ Health Perspect 121:993–1001. doi:10.1289/ehp.1206316 23838256 PMC3764079

[11] Zheng W, Huyan J, Tian Z, Zhang Y, Wen X. 2020. Clinical class 1 integron-integrase gene - A promising indicator to monitor the abundance and elimination of antibiotic resistance genes in an urban wastewater treatment plant. Environ Int 135:105372. doi:10.1016/j.envint.2019.105372 31838265

[12] Shin H, Kim Y, Hur HG. 2023. Differentiation of environment and wastewater treatment plants by core antibiotic resistance genes and aadA2 as indicators in South Korea. Ecol Indic 157:111259. doi:10.1016/j.ecolind.2023.111259

[13] Burch TR, Stokdyk JP, Durso LM, Borchardt MA. 2024. Quantitative microbial risk assessment for ingestion of antibiotic resistance genes from private wells contaminated by human and livestock fecal sources. Appl Environ Microbiol 90:e0162923. doi:10.1128/aem.01629-23 38335112 PMC10952444

[14] Singh G, Vajpayee P, Rani N, Amoah ID, Stenström TA, Shanker R. 2016. Exploring the potential reservoirs of non specific TEM beta lactamase (bla_TEM_) gene in the Indo-Gangetic region: a risk assessment approach to predict health hazards. J Hazard Mater 314:121–128. doi:10.1016/j.jhazmat.2016.04.036 27111425

[15] Pu Q, Zhao LX, Li YT, Su JQ. 2020. Manure fertilization increase antibiotic resistance in soils from typical greenhouse vegetable production bases, China. J Hazard Mater 391:122267. doi:10.1016/j.jhazmat.2020.122267 32062545

[16] Urra J, Alkorta I, Lanzén A, Mijangos I, Garbisu C. 2019. The application of fresh and composted horse and chicken manure affects soil quality, microbial composition and antibiotic resistance. Appl Soil Ecol 135:73–84. doi:10.1016/j.apsoil.2018.11.005

[17] Lau CHF, Tien YC, Stedtfeld RD, Topp E. 2020. Impacts of multi-year field exposure of agricultural soil to macrolide antibiotics on the abundance of antibiotic resistance genes and selected mobile genetic elements. Sci Total Environ 727:138520. doi:10.1016/j.scitotenv.2020.138520 32330714

[18] Yamamoto S, Kitagawa W, Nakano M, Asakura H, Nakayama T, Iwabuchi E, Sone T, Asano K. 2022. Prevalence and characterization of gentamicin resistance genes in Escherichia coli isolates from beef cattle feces in Japan. Curr Microbiol 79:217. doi:10.1007/s00284-022-02913-6 35704076

[19] Javvadi Y, Mohan SV. 2023. Understanding the distribution of antibiotic resistance genes in an urban community using wastewater-based epidemiological approach. Sci Total Environ 868:161419. doi:10.1016/j.scitotenv.2023.161419 36623646

[20] Shi Y, Zhang H, Tian Z, Yang M, Zhang Y. 2018. Characteristics of ARG-carrying plasmidome in the cultivable microbial community from wastewater treatment system under high oxytetracycline concentration. Appl Microbiol Biotechnol 102:1847–1858. doi:10.1007/s00253-018-8738-6 29332216

[21] Uyaguari-Díaz MI, Croxen MA, Luo Z, Cronin KI, Chan M, Baticados WN, Nesbitt MJ, Li S, Miller KM, Dooley D, Hsiao W, Isaac-Renton JL, Tang P, Prystajecky N. 2018. Human activity determines the presence of integron-associated and antibiotic resistance genes in southwestern British Columbia. Front Microbiol 9:852. doi:10.3389/fmicb.2018.00852 29765365 PMC5938356

[22] Huang Y, Wang F, Li Y, Yue C, Zhang Y, Zhou P, Mu J. 2022. Influence of anthropogenic disturbances on antibiotic resistance gene distributions along the Minjiang River in Southeast China. J Environ Manage 323:116154. doi:10.1016/j.jenvman.2022.116154 36095989

[23] Zhang J, Chen M, Sui Q, Tong J, Jiang C, Lu X, Zhang Y, Wei Y. 2016. Impacts of addition of natural zeolite or a nitrification inhibitor on antibiotic resistance genes during sludge composting. Water Res 91:339–349. doi:10.1016/j.watres.2016.01.010 26808292

[24] Yan W, Guo Y, Xiao Y, Wang S, Ding R, Jiang J, Gang H, Wang H, Yang J, Zhao F. 2018. The changes of bacterial communities and antibiotic resistance genes in microbial fuel cells during long-term oxytetracycline processing. Water Res 142:105–114. doi:10.1016/j.watres.2018.05.047 29864646

[25] Lanza VF, Tedim AP, Martínez JL, Baquero F, Coque TM. 2015. The plasmidome of firmicutes: impact on the emergence and the spread of resistance to antimicrobials. Microbiol Spectr 3:LAS–0039. doi:10.1128/microbiolspec.PLAS-0039-2014 26104702

[26] Fatahi-Bafghi M. 2019. Antibiotic resistance genes in the Actinobacteria phylum. Eur J Clin Microbiol Infect Dis 38:1599–1624. doi:10.1007/s10096-019-03580-5 31250336

[27] Sharma P, Kalita MC, Thakur D. 2016. Broad spectrum antimicrobial activity of forest-derived soil actinomycete, Nocardia sp. PB-52. Front Microbiol 7:347. doi:10.3389/fmicb.2016.00347 27047463 PMC4796592

[28] Zhang X, Zhu R, Li W, Ma J, Lin H. 2021. Genomic insights into the antibiotic resistance pattern of the tetracycline-degrading bacterium, Arthrobacter nicotianae OTC-16. Sci Rep 11:15638. doi:10.1038/s41598-021-94840-y 34341372 PMC8329189

[29] Wang Y, Chu L, Ma J, Chi G, Lu C, Chen X. 2022. Effects of multiple antibiotics residues in broiler manure on composting process. Sci Total Environ 817:152808. doi:10.1016/j.scitotenv.2021.152808 34982991

[30] Wang K, Yin D, Sun Z, Wang Z, You SD. 2022. Distribution, horizontal transfer and influencing factors of antibiotic resistance genes and antimicrobial mechanism of compost tea. J Hazard Mater 438:129395. doi:10.1016/j.jhazmat.2022.129395 35803190

[31] Ullmann IF, Tunsjø HS, Andreassen M, Nielsen KM, Lund V, Charnock C. 2019. Detection of aminoglycoside resistant bacteria in sludge samples from Norwegian drinking water treatment plants. Front Microbiol 10:487. doi:10.3389/fmicb.2019.00487 30918503 PMC6424899

[32] Stedtfeld RD, Guo X, Stedtfeld TM, Sheng H, Williams MR, Hauschild K, Gunturu S, Tift L, Wang F, Howe A, Chai B, Yin D, Cole JR, Tiedje JM, Hashsham SA. 2018. Primer set 2.0 for highly parallel qPCR array targeting antibiotic resistance genes and mobile genetic elements. FEMS Microbiol Ecol 94:fiy130. doi:10.1093/femsec/fiy130 30052926 PMC7250373

[33] Ouyang WY, Huang FY, Zhao Y, Li H, Su JQ. 2015. Increased levels of antibiotic resistance in urban stream of Jiulongjiang River, China. Appl Microbiol Biotechnol 99:5697–5707. doi:10.1007/s00253-015-6416-5 25661810

[34] World Health Organization. 2016. Quantitative microbial risk assessment: application for water safety management. Available from: https://www.who.int/publications/i/item/9789241565370

[35] Jiao YN, Zhou ZC, Chen T, Wei YY, Zheng J, Gao RX, Chen H. 2018. Biomarkers of antibiotic resistance genes during seasonal changes in wastewater treatment systems. Environ Pollut 234:79–87. doi:10.1016/j.envpol.2017.11.048 29169020

[36] Zhang A-N, Gaston JM, Dai CL, Zhao S, Poyet M, Groussin M, Yin X, Li L-G, van Loosdrecht MCM, Topp E, Gillings MR, Hanage WP, Tiedje JM, Moniz K, Alm EJ, Zhang T. 2021. An omics-based framework for assessing the health risk of antimicrobial resistance genes. Nat Commun 12:4765. doi:10.1038/s41467-021-25096-3 34362925 PMC8346589

[37] Zhang Z, Zhang Q, Wang T, Xu N, Lu T, Hong W, Penuelas J, Gillings M, Wang M, Gao W, Qian H. 2022. Assessment of global health risk of antibiotic resistance genes. Nat Commun 13:1553. doi:10.1038/s41467-022-29283-8 35322038 PMC8943045

[38] Panagiotou CF, Stefan C, Papanastasiou P, Sprenger C. 2023. Quantitative microbial risk assessment (QMRA) for setting health-based performance targets during soil aquifer treatment. Environ Sci Pollut Res Int 30:14424–14438. doi:10.1007/s11356-022-22729-y 36151439

[39] Park H-S, Lee YK, Kim H, Lee K. 2017. Suggestion of a method to assess the risk level of agricultural works considering work posture and working time. J Ergon Soc Korea 36:601–607. doi:10.5143/JESK.2017.36.5.601

[40] Lee I, Kim J. 2012. Survey of the characteristics of the symptoms of musculoskeletal disorders among farmers of fruits and vegetables. J Korean Soc Saf 27:144–150. doi:10.14346/JKOSOS.2012.27.6.144

[41] Callahan BJ, McMurdie PJ, Rosen MJ, Han AW, Johnson AJA, Holmes SP. 2016. DADA2: high-resolution sample inference from Illumina amplicon data. Nat Methods 13:581–583. doi:10.1038/nmeth.3869 27214047 PMC4927377

[42] Wang Q, Garrity GM, Tiedje JM, Cole JR. 2007. Naive Bayesian classifier for rapid assignment of RRNA sequences into the new bacterial taxonomy. Appl Environ Microbiol 73:5261–5267. doi:10.1128/AEM.00062-07 17586664 PMC1950982

[43] Caporaso JG, Kuczynski J, Stombaugh J, Bittinger K, Bushman FD, Costello EK, Fierer N, Peña AG, Goodrich JK, Gordon JI, et al.. 2010. QIIME allows analysis of high-throughput community sequencing data. Nat Methods 7:335–336. doi:10.1038/nmeth.f.303 20383131 PMC3156573

[44] Segata N, Izard J, Waldron L, Gevers D, Miropolsky L, Garrett WS, Huttenhower C. 2011. Metagenomic biomarker discovery and explanation. Genome Biol 12:R60. doi:10.1186/gb-2011-12-6-r60 21702898 PMC3218848

[45] Antwi-Agyei P, Biran A, Peasey A, Bruce J, Ensink J. 2016. A faecal exposure assessment of farm workers in Accra, Ghana: a cross sectional study. BMC Public Health 16:587. doi:10.1186/s12889-016-3266-8 27423694 PMC4947311

