# Supplementary material for: Quantitative microbial risk assessment of antibiotic resistance genes and mobile genetic elements in orchard soils across South Korea
Source: Appl Environ Microbiol. 2025 Dec 18;92(1):e02260-25. doi: 10.1128/aem.02260-25 (PMC12838445; doi:10.1128/aem.02260-25)
Supplement: Supplemental figures and tables — Figures S1 to S4; Tables S1 and S2. [file aem.02260-25-s0001.docx]

**Quantitative Microbial Risk Assessment of Antibiotic Resistance Genes and Mobile Genetic Elements in Orchard Soils of South Korea**

Raan Shin^1^, Seunggyun Han^1^, Jaeyoung Ro^1^, Sujin Lee^1^, Hor-Gil Hur^1*^ and Hanseob Shin^2,3*^

^1^School of Environment and Energy Engineering, Gwangju Institute of Science and Technology (GIST), Gwangju, 61005, Republic of Korea

^3^Center for Health Effects of Environmental Contamination, University of Iowa, W195 Chemistry Building, University of Iowa, Iowa City, Iowa, United States

^4^State Hygienic Laboratory, University of Iowa, Coralville, Iowa, United States

***Corresponding authors**

**Hanseob Shin, Ph.D.**

E-mail: hanshin@uiowa.edu, Tel: +1 (319) 259-3517;

**Hor-Gil Hur, Ph.D., Professor**

E-mail: hghur@gist.ac.kr, [Tel: +82-62-715-2437](Tel:+82-62-715-2437), Fax: +82-62-715-2434

**
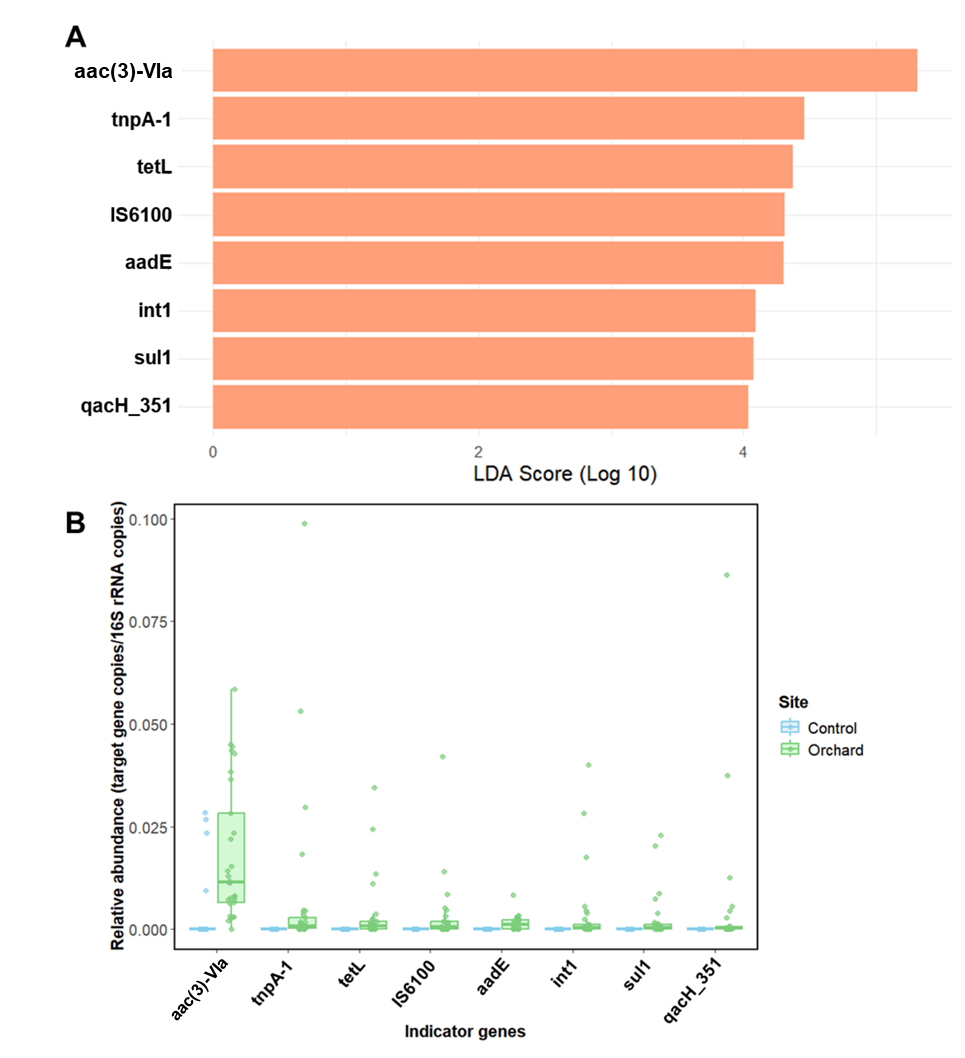
**

**Figure S1. Core antibiotic resistance genes (ARGs) and mobile genetic elements (MGEs) identified by Llinear discriminant analysis effect size (LEfSe) and their relative abundances in orchard and control (non-orchard) soil.** (A) Linear discriminant analysis (LDA) scores of core ARGs and MGEs significantly enriched in orchard soils compared to control soils, as identified by LEfSe. LDA score cutoff of 4 was applied to determine significant differences. Higher LDA scores indicate stronger discriminatory power between groups. (B) Relative abundances of the eight core indicator genes (normalized to 16S rRNA gene copies) in orchard and control soils. Boxplots represent the distribution of gene abundances, with individual points showing sample-level values. ARGs and MGEs were significantly more enriched in orchard soils compared to controls. (Wilcoxon test, *p* < 0.05)

**
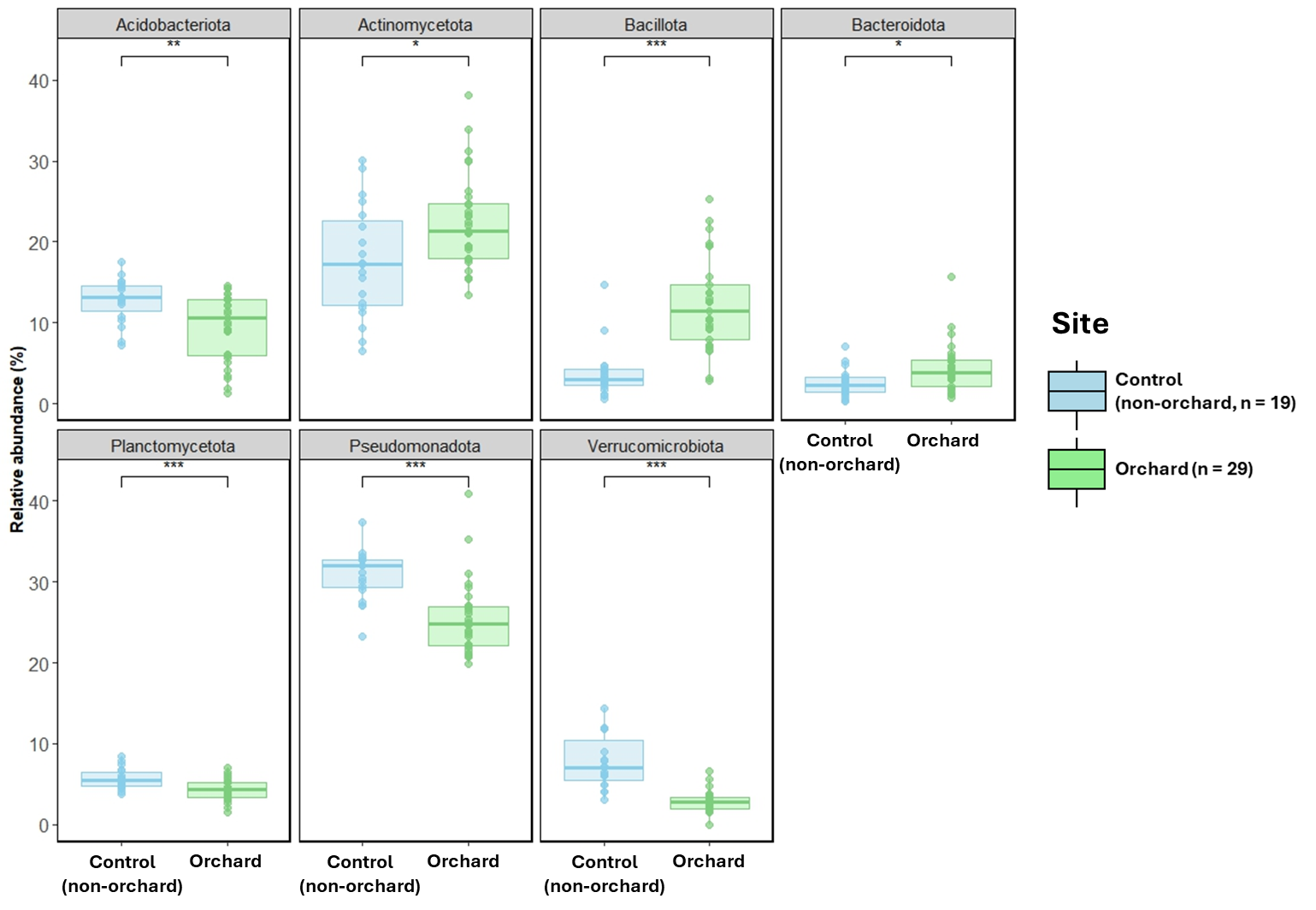
**

**Figure S2.** Comparison of relative abundances of dominant bacterial phyla in orchard and control soils. Boxplots show the distribution of relative abundances for the seven most dominant phyla across orchard (green) and control (blue) soils. Statistical comparisons were performed using the Wilcoxon matched-pair test. (*, *p* < 0.05; **, *p* < 0.01; ***, *p* < 0.001, Wilcoxon test)

**
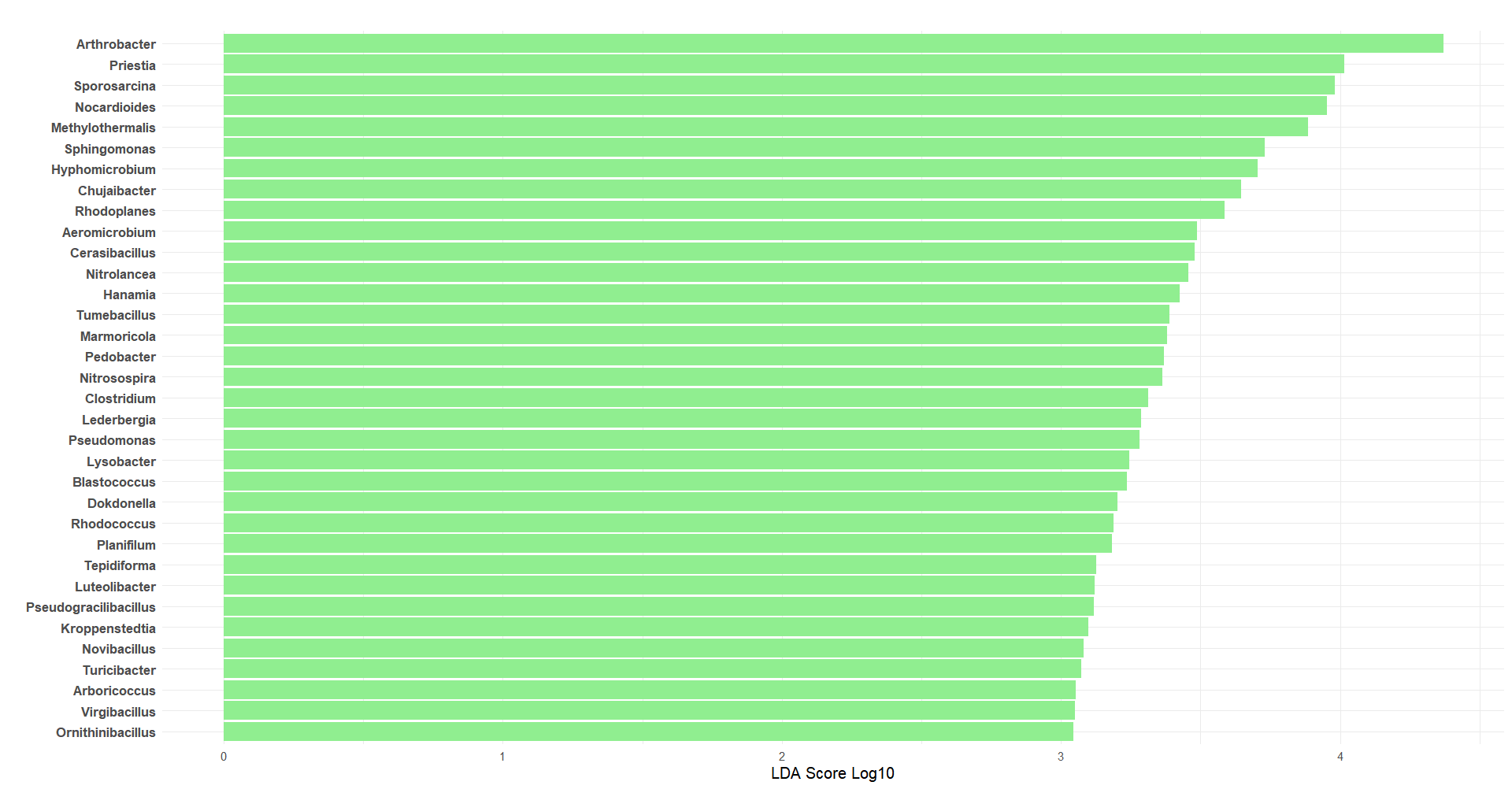
**

**Figure S3.** The Linear Discriminant Analysis Effect Size (LEfSe) method to identify the most differentially enriched bacterial genera between orchard and control samples. Genera within the orchard samples showed significant differential abundance. (LDA score > 3)

**
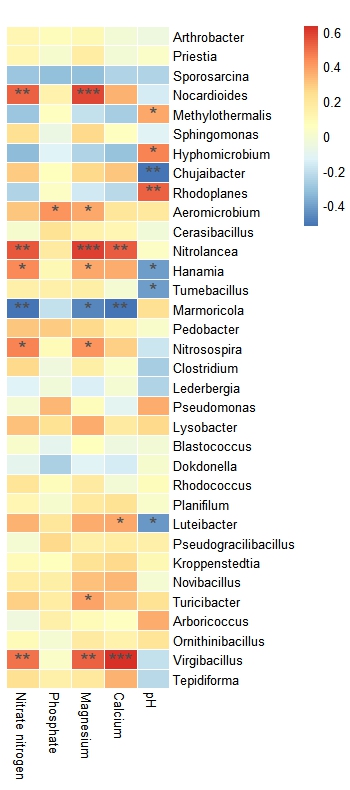
**

**Figure S4.** Heatmap of correlations between soil physicochemical properties and bacterial genera enriched in orchard soils. Spearman correlation analysis was performed to examine relationships between soil parameters and bacterial genera. The color scale represents correlation coefficients (red = positive correlation; blue = negative correlation), and significance is indicated by asterisks (*: *p* < 0.05, **: *p* < 0.01, ***: *p* < 0.001).

**Table S1.** Relative abundance of the top 5 predominant bacteria at the phylum level in orchard and control samples.

| Control | | Orchard | |
| --- | --- | --- | --- |
| Phylum | Relative abundance (%) | Phylum | Relative abundance (%) |
| Pseudomonadota | 34.3 (23.3-37.3) | Pseudomonadota | 27.9 (19.9-40.9) |
| Actinomycetota | 19.5 (6.54-30.1) | Actinomycetota | 24.6 (13.4-38.2) |
| Acidobacteriota | 14.2 (7.26-17.6) | Bacillota | 13.4 (2.9-25.3) |
| Verrucomicrobiota | 8.59 (3.1-14.3) | Acidobacteriota | 10.4 (1.34-14.6) |
| Planctomycetota | 6.32 (3.81-8.51) | Bacteroidota | 4.77 (1.07-15.7) |

**Table S2.** Information on the sampling sites in this study

| **Site** | **Sample** | **Province** | **City** | **GPS** | **Fruit** |
| --- | --- | --- | --- | --- | --- |
| Orchard | GN1 | Gyeongsangnam-do | Hadong | 35°05'20"N 127°43'05"E | Pear |
|  | GN3 | Gyeongsangnam-do | Jinju | 35°12'35"N 128°07'06"E | Pear |
|  | GN5 | Gyeongsangnam-do | Miryang | 35°28'18"N 128°43'41"E | Grape |
|  | GB6 | Gyeongsangbuk-do | Gyeongsan | 35°48'38"N 128°48'47"E | Grape |
|  | GB7 | Gyeongsangbuk-do | Gyeongsan | 35°50'02"N 128°48'45"E | Grape |
|  | GB8 | Gyeongsangbuk-do | Gunwi | 36°16'00"N 128°29'48"E | Apple |
|  | GB10 | Gyeongsangbuk-do | Yecheon | 36°44'33"N 128°26'28"E | Apple |
|  | GW13 | Gangwon-do | Jeongseon | 37°11'40"N 128°42'16"E | Apple |
|  | GW14 | Gangwon-do | Wonju | 37°19'30"N 127°55'03"E | Pear |
|  | GW15 | Gangwon-do | Hongcheon | 37°34'06"N 127°49'17"E | Pear |
|  | GG17 | Gyeonggi-do | Yangpyeong | 37°32'17"N 127°45'53"E | Apple |
|  | GG19 | Gyeonggi-do | Icheon | 37°11'49"N 127°29'28"E | Pear |
|  | GG20 | Gyeonggi-do | Anseong | 36°56'14"N 127°15'41"E | Grape |
|  | GG21 | Gyeonggi-do | Anseong | 36°56'22"N 127°15'34"E | Grape |
|  | CN22 | Chungcheongnam-do | Cheonan | 36°55'54"N 127°12'05"E | Grape |
|  | CN23 | Chungcheongnam-do | Cheonan | 36°54'39"N 127°13'51"E | Grape |
|  | CN27 | Chungcheongnam-do | Geumsan | 36°13'09"N 127°29'53"E | Grape |
|  | CN28 | Chungcheongnam-do | Geumsan | 36°10'10"N 127°31'35"E | Pear |
|  | CB11 | Chungcheongbuk-do | Danyang | 36°53'28"N 128°17'41"E | Apple |
|  | CB25 | Chungcheongbuk-do | Cheongju | 36°41'45"N 127°28'43"E | Apple |
|  | CB29 | Chungcheongbuk-do | Yeongdong | 36°08'16"N 127°44'31"E | Apple |
|  | JB31 | Jeonbuk-do | Muju | 36°01'49"N 127°42'19"E | Apple |
|  | JB32 | Jeonbuk-do | Jangsu | 35°41'46"N 127°34'09"E | Apple |
|  | JB33 | Jeonbuk-do | Jinan | 35°48'09"N 127°25'20"E | Apple |
|  | JB35 | Jeonbuk-do | Jinan | 35°40'50"N 127°23'24"E | Apple |
|  | JN36 | Jeollanam -do | Gokseong | 35°17'41"N 127°10'06"E | Apple |
|  | JN38 | Jeollanam -do | Damyang | 35°13'28"N 126°59'02"E | Grape |
|  | JN39 | Jeollanam -do | Damyang | 35°13'39"N 126°59'07"E | Grape |
|  | JN40 | Jeollanam -do | Jangseong | 35°14'17"N 126°48'56"E | Pear |
| Control | GC1 | Jeonbuk-do | Kochang | 35°29'50"N 126°34'16"E | Provincial Park |
|  | GC2 | Jeonbuk-do | Kochang | 35°29'50"N 126°34'16"E | Provincial Park |
|  | GC3 | Jeonbuk-do | Kochang | 35°29'46"N 126°34'44"E | Provincial Park |
|  | GC4 | Jeonbuk-do | Kochang | 35°29'46"N 126°34'44"E | Provincial Park |
|  | YG1 | Jeollanam-do | Yeong-gwang | 35°11'57"N 126°33'03"E | Provincial Park |
|  | YG2 | Jeollanam-do | Yeong-gwang | 35°11'57"N 126°33'03"E | Provincial Park |
|  | YG3 | Jeollanam-do | Yeong-gwang | 35°11'30"N 126°32'41"E | Provincial Park |
|  | YG4 | Jeollanam-do | Yeong-gwang | 35°11'30"N 126°32'41"E | Provincial Park |
|  | BP1 | Jeollanam-do | Damyang | 35°18'50"N 126°52'31"E | Mountain |
|  | BP2 | Jeollanam-do | Damyang | 35°18'50"N 126°52'31"E | Mountain |
|  | BP3 | Jeollanam-do | Damyang | 35°18'46"N 126°52'28"E | Mountain |
|  | BP4 | Jeollanam-do | Jangseong | 35°20'04"N 126°52'20"E | Mountain |
|  | BP5 | Jeollanam-do | Jangseong | 35°20'03"N 126°52'22"E | Mountain |
|  | BT1 | Jeollanam-do | Jangseong | 35°16'54"N 126°50'30"E | Mountain |
|  | BT2 | Jeollanam-do | Jangseong | 35°17'02"N 126°50'30"E | Mountain |
|  | BT3 | Jeollanam-do | Jangseong | 35°16'50"N 126°50'31"E | Mountain |
|  | BT4 | Jeollanam-do | Jangseong | 35°17'37"N 126°50'21"E | Mountain |
|  | SI1 | Jeollanam-do | Damyang | 35°18'02"N 126°53'40"E | Mountain |
|  | SI2 | Jeollanam-do | Damyang | 35°18'02"N 126°53'40"E | Mountain |
