# Supplementary material for: Quantitative microbial risk assessment of antibiotic resistance genes and mobile genetic elements in orchard soils across South Korea
Source: Appl Environ Microbiol. 2025 Dec 18;92(1):e02260-25. doi: 10.1128/aem.02260-25 (PMC12838445; doi:10.1128/aem.02260-25)
Supplement: Supplemental text — Supplemental results. [file aem.02260-25-s0002.docx]

**Supporting Information**

**Quantitative Microbial Risk Assessment of Antibiotic Resistance Genes and Mobile Genetic Elements in Orchard Soils of South Korea**

Raan Shin^1^, Seunggyun Han^1^, Jaeyoung Ro^1^, Sujin Lee^1^, Song-Hee Ryu^2^ Hor-Gil Hur^1*^ and Hanseob Shin^3,4*^

^1^School of Environment and Energy Engineering, Gwangju Institute of Science and Technology (GIST), Gwangju, 61005, Republic of Korea

^2^Residual Agrochemical Assessment Division, National Institute of Agricultural Sciences, Wanju-gun, South Korea

^3^Center for Health Effects of Environmental Contamination, University of Iowa, W195 Chemistry Building, University of Iowa, Iowa City, Iowa, United States

^4^State Hygienic Laboratory, University of Iowa, Coralville, Iowa, United States

***Corresponding authors**

**Hanseob Shin, Ph.D.**

E-mail: hanshin@uiowa.edu, Tel: +1 (319) 259-3517

**Hor-Gil Hur, Ph.D., Professor**

E-mail: hghur@gist.ac.kr, Tel: +82-62-715-2437, Fax: +82-62-715-2434

**Influence of soil physicochemical characteristics on bacterial communities and ARGs**

To investigate the soil environments in orchard soils, we analyzed the concentrations of four soil ions (Mg^2+^, Ca^2+^, NO^3-^, PO_4_^3-^) and pH (**Figure S5**). The concentrations of NO^3-^ and Mg^2+^ were significantly higher in orchard soils compared to control soils (*p* < 0.001), and the pH was also significantly increased in orchard soils (*p* < 0.01). In contrast, there were no significant differences in Ca^2+^ and PO_4_^3-^.

Physiochemical characteristics are known to influence the distribution of ARGs within the soil, and our results show that orchard soils exhibited distinct physiochemical characteristics compared to control soils^1^**.** These agricultural inputs have been reported to increase the availability of nutrients, as seen in previous studies where manure treatments led to higher levels of available nitrogen^2^ and magnesium^3^. Manure application has been associated with increased soil pH levels^4^, which is known to influence microbial community structure by altering both diversity and the relative abundance of specific taxa^5^. Together, such practices may also induce changes in the soil resistome, as manure application can directly introduce ARGs into the soil or exert selective pressure on microbial communities through residual antibiotics^6^. Chemical fertilizers have also been associated with increased ARG abundance^7^, suggesting that fertilizer components can affect ion composition in soil, which can change microbial communities and ARG distribution.

**Correlation of soil physiochemical parameters and bacterial communities in orchard**

We analyzed the correlations between soil physiochemical parameters (pH, Mg²⁺, Ca²⁺, NO₃⁻, and PO₄³⁻) and bacterial communities in orchard soils. Spearman correlation analysis revealed that among the 34 genera differentially enriched in orchard soils, 14 genera exhibited significant correlations with environmental factors (**Figure S5**). Specifically, NO₃⁻ was positively correlated with 5 genera and negatively correlated with one genus, while PO₄³⁻ showed a positive correlation with only one genus. Moreover, Mg²⁺ showed positive correlations with seven genera and a negative correlation with one genus, and Ca²⁺ was positively correlated with three genera but negatively correlated with one genus. pH was positively correlated with three genera and negatively correlated with four genera, indicating that among the environmental factors, pH exhibited the most extensive negative associations with bacterial genera.

Anthropogenic activities, which has been reported to alter soil resistome can also induce shifts in microbial community structure^8^, and increase the load of ARB^9^. Among environmental factors, pH is recognized as a key driver of bacterial community variation, and our data show that pH exhibited the negative correlation. In addition, several genera showed significant correlations with NO₃⁻ and Mg²⁺, both of which were notably elevated in orchard soils. Previous studies have also reported positive correlations between nitrate levels and ARG abundance^10^, suggesting that nutrient enrichment may influence microbial communities and resistance gene profiles. Notably, *Nitrolancea*, which was positively correlated with two key ions (Mg²⁺, and NO₃⁻), also identified positive associations with 4 core ARGs, suggesting its potential role in the proliferation of resistance genes under nutrient-enriched conditions (**Figure S4**). Similarly, *Nocardioides*, which showed positive correlations with Mg²⁺, and NO₃⁻, was significantly associated with the *tnpA-2*, and *Aeromicrobium*, also positively correlated with Mg²⁺, exhibited a similar relationship with *tnpA-2* (**Figure S4**.). The frequent detection of nitrite-oxidizing bacteria (i.e. *Nitrolancea* spp.) in orchard soils is consistent with the widespread use of ammonium-based fertilizers (e.g., urea) in East Asia, in particular South Korea. Such fertilizers provide ammonia as a nitrogen source, which is subsequently converted to nitrite and nitrate through microbial nitrification, thereby supporting the activity of nitrite-oxidizing bacteria and shaping nitrogen cycling within soils^11^. These results suggest that long-term application of fertilizers or manure may contribute to soil eutrophication, thereby promoting the enrichment of specific bacterial genera that are closely linked to ARGs and MGEs.

**Bacterial community structure in orchard soils**

At the genus level, *Arthrobacter* sp. was the most differentially enriched taxon in orchard soils. This genus, belonging to Actinomycetota, is notable for its ability to degrade tetracycline^42^, linking its enrichment to the selective pressure from oxytetracycline application in orchards. Another enriched genus, *Priestia* sp., previously isolated from pear orchard soils in China, exhibited strong inhibitory activity against *Erwinia amylovora*, the causative agent of fire blight^43^. However, this genus also exhibited resistance to multiple antibiotics, including ampicillin, erythromycin, penicillin, and tetracycline^43^, reflecting the potential role of beneficial orchard-associated bacteria as ARG reservoirs. Within the phylum Pseudomonadota, *Sphingomonas* sp. is known to harbor multiple efflux pumps that enhance survival rate under antimicrobial stress^44^. Its enrichment in orchard soils suggests that efflux-mediated tolerance may contribute to ARG persistence and dissemination in agricultural environments. Our microbiome analysis can show that the enrichment of such specific bacterial genera in orchard soils can be shaped by agricultural practices, including antibiotic use and manure application.

**References**

(1) Xiao, R.; Huang, D.; Du, L.; Song, B.; Yin, L.; Chen, Y.; Gao, L.; Li, R.; Huang, H.; Zeng, G. Antibiotic Resistance in Soil-Plant Systems: A Review of the Source, Dissemination, Influence Factors, and Potential Exposure Risks. *Sci. Total Environ.* **2023**, *869*, 161855. https://doi.org/10.1016/j.scitotenv.2023.161855.

(2) Li, J.; Xin, Z.; Zhang, Y.; Chen, J.; Yan, J.; Li, H.; Hu, H. Long-Term Manure Application Increased the Levels of Antibiotics and Antibiotic Resistance Genes in a Greenhouse Soil. *Appl. Soil Ecol.* **2017**, *121*, 193–200. https://doi.org/10.1016/j.apsoil.2017.10.007.

(3) Sienkiewicz, S.; Krzebietke, S.; Wojnowska, T.; arczyñski, P.; Omilian, M. Effect of Long-Term Differentiated Fertilization with Farmyard Manure and Mineral Fertilizers on the Content of Available Forms of P, K and Mg in Soil. *J. Elementol* **2009**, *14* (4), 779–786.

(4) Whalen, J. K.; Chang, C.; Clayton, G. W.; Carefoot, J. P. Cattle Manure Amendments Can Increase the PH of Acid Soils. *Soil Sci. Soc. Am. J.* **2000**, *64* (3), 962–966. https://doi.org/10.2136/sssaj2000.643962x.

(5) Xiong, R.; He, X.; Gao, N.; Li, Q.; Qiu, Z.; Hou, Y.; Shen, W. Soil PH Amendment Alters the Abundance, Diversity, and Composition of Microbial Communities in Two Contrasting Agricultural Soils. *Microbiol Spectr* **2024**, *12* (8), 19. https://doi.org/10.1128/spectrum.04165-23.

(6) Xie, W. Y.; Shen, Q.; Zhao, F. J. Antibiotics and Antibiotic Resistance from Animal Manures to Soil: A Review. *Eur. J. Soil Sci.* **2018**, *69* (1), 181–195. https://doi.org/10.1111/ejss.12494.

(7) Wang, F.; Xu, M.; Stedtfeld, R. D.; Sheng, H.; Fan, J.; Liu, M.; Chai, B.; Soares De Carvalho, T.; Li, H.; Li, Z.; Hashsham, S. A.; Tiedje, J. M. Long-Term Effect of Different Fertilization and Cropping Systems on the Soil Antibiotic Resistome. *Environ Sci Technol* **2018**, *52* (22), 13037–13046. https://doi.org/10.1021/acs.est.8b04330.

(8) Zalewska, M.; Błażejewska, A.; Czapko, A.; Popowska, M. Antibiotics and Antibiotic Resistance Genes in Animal Manure – Consequences of Its Application in Agriculture. *Front Microbiol* **2021**, *12*, 610656. https://doi.org/10.3389/fmicb.2021.610656.

(9) Zhu, Y. G.; Johnson, T. A.; Su, J. Q.; Qiao, M.; Guo, G. X.; Stedtfeld, R. D.; Hashsham, S. A.; Tiedje, J. M. Diverse and Abundant Antibiotic Resistance Genes in Chinese Swine Farms. *Proc Natl Acad Sci U S A* **2013**, *110* (9), 3435–3440. https://doi.org/10.1073/pnas.1222743110.

(10) Xu, K.; Liu, X.; Pang, L.; Yue, Y.; Chatzisymeon, E.; Yang, P. Response Behavior of Antibiotic Resistance Genes and Human Pathogens to Slope Gradient and Position: An Environmental Risk Analysis in Sloping Cultivated Land. *Sci. Total Environ.* **2023**, *905*, 166994. https://doi.org/10.1016/j.scitotenv.2023.166994.

(11) Huang, L.; Chakrabarti, S.; Cooper, J.; Perez, A.; John, S. M.; Daroub, S. H.; Martens-Habbena, W. Ammonia-Oxidizing Archaea Are Integral to Nitrogen Cycling in a Highly Fertile Agricultural Soil. *ISME COMMUN* **2021**, *1* (1), 19. https://doi.org/10.1038/s43705-021-00020-4.
